# Periostin Promotes Sarcoma Growth by Promoting Tumor-Associated Macrophage Migration and Differentiation

**DOI:** 10.1158/2767-9764.CRC-25-0301

**Published:** 2025-12-26

**Authors:** Jin-Fen Xiao, Kristin Ishaya, Emily Y. Ko, Annaliese Fowler, Marina T. Broz, Jlenia Guarnerio

**Affiliations:** 1 Department of Radiation Oncology, Cedars-Sinai Medical Center, Los Angeles, California.; 2 Department of Genetics, University of Texas MD Anderson Cancer Center, Houston, Texas.; 3 Department of Biomedical Sciences, Cedars-Sinai Medical Center, Los Angeles, California.; 4 David Geffen Medical School, Department of Medicine, University of California Los Angeles, Los Angeles, California.

## Abstract

Soft-tissue sarcomas (STS) are characterized by abundant extracellular matrix (ECM) deposition, yet the functional contribution of specific ECM components remains poorly understood. In this study, we identify periostin (POSTN), a matricellular protein, as a regulator of sarcoma progression and the tumor immune microenvironment. Analysis of human sarcoma datasets revealed that high *POSTN* expression correlates with poor prognosis and elevated expression of ECM-related and myeloid cell–associated genes. In murine genetic models of sarcoma, tumors expressing high levels of *Postn* displayed enhanced expression of ECM genes and monocyte-recruiting cytokines. Functional silencing of *Postnin vivo* reduced tumor growth without altering tumor cell proliferation or intrinsic signaling pathways, suggesting a noncell-autonomous mechanism. Instead, *Postn*-deficient tumors showed increased infiltration of CD4^+^ and CD8^+^ T cells and reduced proportions of immunosuppressive myeloid cells, including tumor-associated macrophages (TAM). Single-cell RNA sequencing revealed that *Postn *silencing reprograms the myeloid compartment, increasing IFN-responsive and proinflammatory subsets. Mechanistically, recombinant POSTN promoted monocyte migration and maturation into macrophages *in vitro*, supporting its role as a chemoattractant and differentiation cue. Therapeutic neutralization of POSTN partially recapitulated the immunologic remodeling but was insufficient to reduce tumor burden as monotherapy.

**Significance:**

Our findings position POSTN as a key stromal regulator in STS, linking tumor-derived ECM components to immune evasion via myeloid cell recruitment and education. These results open avenues for targeting POSTN as an adjuvant strategy to enhance the efficacy of immunotherapies and overcome the immune-excluded phenotype of sarcoma.

## Introduction

Soft-tissue sarcomas (STS) are a diverse group of malignancies typically treated with surgery and radiotherapy as primary interventions. Although these approaches are effective initially, disease recurrence and metastasis remain major clinical challenges (1). Anthracycline-based chemotherapy, such as doxorubicin, is commonly employed as a second-line treatment for various sarcoma subtypes, including those with undifferentiated mesenchymal cells; the therapeutic efficacy of chemotherapy is often limited by tumor cell resistance, either intrinsic or acquired, which contributes to tumor relapse (2, 3). No additional curative options are currently available for patients (4–6).

Emerging evidence highlights cellular and molecular components of the tumor microenvironment (TME) as additional critical players in tumor progression (7–9). Sarcoma cells, due to their mesenchymal nature, produce and deposit large amounts of extracellular matrix (ECM) proteins, which are associated with poor overall survival (10, 11). Moreover, ECM elements can shield tumor cells from chemotherapy and promote relapse (12). For example, previous studies reported that ECM stiffness and the interactions between multiple ECM components with integrins, such as β1-integrin, activate pathways like ILK/PI3K/AKT and FAK, which enhance stemness, proliferation, and resistance to therapy (13). Despite this, the functional roles of specific ECM components and the molecular mechanisms of action in sarcoma remain underexplored.

Myeloid cells, encompassing both monocytes and tumor-associated macrophages (TAM), are the most abundant immune components in sarcomas (14, 15). TAMs can play pro- or antitumorigenic roles based on their activation state. From one side, TAMs have been shown to contribute to tumor progression in multiple ways, including by creating an immunosuppressive microenvironment (16), favoring angiogenesis, and releasing metabolites that have recently emerged as drivers of drug resistance mechanisms (17–19). Accordingly, blocking TAM accumulation and survival in the tumor mass (e.g., by blocking the CSF1/CSF1R axis) has been tested as a potential immunotherapeutic intervention (20–22). On the other side, TAMs can have antitumorigenic properties and potentially kill tumor cells through the activation of adaptive immune responses by releasing IFN and inflammatory factors or directly engulfing live tumor cells in a process described as entosis (23). TAMs’ phagocytic activities are even potentiated by the presence of tumor-specific antibodies [called antibody-dependent cellular phagocytosis (ADCP); ref. 24]. In this respect, newer cellular therapies have assessed the potential engineering of macrophages to directly target tumor cells, exploiting the macrophages’ unique capability to infiltrate the inner tumor parenchyma and to reach even hypoxic and necrotic areas of the tumor mass (25).

In this study, we investigated whether and how specific ECM proteins influence macrophage recruitment and activation in sarcoma. We focused on periostin (POSTN), a protein recently associated with poor prognosis in patients with sarcoma (26). Using a syngeneic sarcoma mouse model, we found that *Postn* is highly expressed in sarcoma cells and that its silencing can reduce tumor growth. Mechanistically, *Postn* silencing did not significantly alter the gene expression profile nor the proliferation rate of the tumor cells themselves but rather induced substantial changes in the immune TME. Specifically, we observed an increase in monocytes and T cells within the tumor accompanied by a proportional decrease in TAMs. *In vitro* functional assays further demonstrated that POSTN acts as a chemoattractant for bone marrow–derived monocytes, promoting their recruitment to the tumor, in which they subsequently differentiate into TAMs. Importantly, our preclinical data suggest that targeting POSTN can effectively reshape the immune landscape of the TME. This points to the possibility of combining POSTN inhibition with the standard of care or with other immunotherapies that have previously been unsuccessful in the context of sarcoma, such as immune checkpoint inhibitors.

## Materials and Methods

### Patient analysis

Patient survival analysis was performed with The Cancer Genome Atlas (TCGA) datasets and using the online survival analysis tool KM-plotter with default cutoffs for *POSTN* expression (27). Correlation between *POSTN* expression and other relevant genes was determined using cBioPortal (https://www.cbioportal.org/). For differential expression and gene set enrichment analysis (based on log fold-changes), patients were stratified by *POSTN* mRNA expression z-score >1 or < −1. A maximum of 15 enriched gene sets are shown per condition.

### Mice

C57BL/6J wild type (#00064) and p53 knockout (KO; #002101) were purchased from The Jackson Laboratory and used to generate syngeneic tumor cell lines p53^KO^ Ccne1^+^ and p53^KO^ Vgll3^+^ from female mice. 8-week-old female mice were used as tumor recipients for all experiments. Tumor burden did not exceed 1.5 cm^3^. Maximal tumor burden and all other aspects of animal experiments were performed in accordance with the guidelines of the Cedars-Sinai Medical Center Institutional Animal Care and Use Committee. For the experiment with anti-POSTN antibody treatment, the blocking antibody (OC-20) was purchased from AdipoGen (AG-20B-6000YPF) and injected intratumorally at a concentration of 50 μg/mouse every 3 days for a total of three injections.

### Mouse mesenchymal stromal cell isolation, maintenance, and *in vivo* tumorigenesis

Subcutaneous sarcomas were generated by injecting sarcoma cells in Matrigel. Briefly, long bones were collected from p53^KO^ mice, crushed, and digested with collagenase II (1 mg/mL) for 1 hour at 37°C on a shaker. Recovered cells were stained and FACS-sorted (CD45^−^CD31^−^Ter119^−^Sca1^+^PDGFRα^+^) to obtain mesenchymal stem cells and cultured in complete MesenCult medium (STEMCELL Technologies). Mouse mesenchymal stromal cells (MSC) were maintained in a humidified chamber with 5% CO_2_, with half of the medium changed every 3 days. After 7 days in culture, cells formed visible colony-forming units-fibroblasts; after this point, cells were periodically split at 80% confluency. To generate sarcoma cells, mesenchymal cells were transduced for the stable expression of *Ccne1* or *Vgll3* and red fluorescent protein (see below for plasmid generation). After transduction, cells were cultured in DMEM with 10% FBS. Cells were transplanted subcutaneously in mice for two consecutive rounds, as previously described (28). The stable cells were collected back from the second recipients, expanded in culture in DMEM with 10% FBS, and assessed by qRT-PCR and/or Western blot. These cells were used for *in vitro* experiments and *in vivo* tumorigenesis assays. Tumor volume was monitored every 2 to 3 days by caliper measurement. Tumor volume was calculated using the standard formula (width × length × length/2).

### Tissue dissociation

Tumors were harvested, minced, and then enzymatically and mechanically digested using the *37C_m_TDK_2* protocol on the Miltenyi gentleMACS with the Tumor Dissociation Kit for Mouse (Miltenyi Biotec). Cell suspensions were washed in Cell Staining Buffer (BioLegend) and filtered through 70 μm strainers (Bioland Scientific LLC). Red blood cells were lysed with ammonium-chloride-potassium (ACK) buffer.

### Single-cell RNA sequencing and analysis

Single-cell suspensions from mouse tumors were generated as above. CD11b^+^ myeloid cells were FACS-sorted. Cells from five individual mice were each tagged with a unique TotalSeq-B Hashtag reagent (BioLegend)—an oligonucleotide barcode conjugated to antibodies against universally expressed cell surface proteins. Final cell suspensions were washed three times in PBS, filtered through 40 μm Bel-Art Flowmi strainers (Bel-Art/SP Scienceware), viability verified by trypan blue staining, counted, and pooled into a single sample at a concentration of 1,000 cells/μL. The cell suspension was loaded into the Chromium Controller or Chromium X (10x Genomics) for single-cell mRNA capture using the Next GEM 3′ version 3.1 kit. Reverse transcription and library preparation steps were performed in accordance with manufacturer protocols. Completed libraries were sequenced at the Cedars-Sinai Applied Genomics Core Facility.

### Processing of single-cell RNA sequencing datasets

To produce gene expression count matrices, FASTQ reads were aligned and counted with the Cell Ranger count pipeline (10x Genomics). In the case of mouse tumors, reads were aligned against a custom reference genome that included the sequence for the mRFP transgene expressed by the malignant cells; otherwise, default Cell Ranger references were used. For all further downstream processing, Seurat version 5 was used.

Counts were normalized and variance-stabilized by *SCTransform* with the method parameter “*glmGamPoi*.” Dimensionality reduction, visualization, and initial clustering were performed using *RunPCA, RunTSNE*, *FindNeighbors*, and *FindClusters*. Differentially expressed genes were calculated using *FindAllMarkers*. Sample identity was demultiplexed using *HTODemux*. Cells were labeled as dead, ambient, or otherwise failing quality check if the percentage of mitochondrial reads was greater than 15%, belonged to a cell cluster in which the top 10 differentially expressed genes were mostly mitochondrial transcripts or were marked as a doublet by either *HTODemux* or *scDblFinder*. Dimensionality reduction, clustering, and visualization were repeated on the filtered dataset.

### Bulk RNA sequencing analysis

Transcripts were quantified with *salmon*, and differential expression analysis was conducted in *DESeq2*. For heatmaps, genes exhibiting an absolute log fold change greater than 1 were ranked by *P* value, and the top genes were plotted. Gene set enrichment analysis (GSEA) was conducted on genes ranked by fold-change, using the *clusterProfiler* package for R and the Mouse MSigDB Hallmarks gene set collection. Significantly enriched gene sets (Benjamini-Hochberg adjusted *P* values < 0.05) were reported.

### Generation of retrovirus, lentivirus, knockdown, KO, and overexpressing cells

The retroviral vector pCMMP-MCS-IRES-mRFP (Addgene, #36972) was used for the overexpression of the *Ccne1* and *Vgll3* genes. The genes were amplified from the cDNA of mouse mesenchymal cells and cloned into the retroviral vector using the Gibson Assembly kit (New England Biolabs). The expression of the transgene was assessed by qRT-PCR. Short hairpin RNAs (shRNA) were cloned into the pLKO.1 lentiviral vector (Addgene), following Addgene’s instructions. The shRNA sequences were designed according to the following program provided by the Broad Institute GPP Web Portal: https://portals.broadinstitute.org/gpp/public/seq/search.

All the viral particles were produced in HEK 293T cells, which were cotransfected with the specific viral vector and packaging-expressing plasmids: pECO for the retroviral vectors and VSV-G, REV, and dR8.74 for the lentiviral vectors. Transfection of the cells was performed using Polyplus (Sartorius), according to the manufacturer’s instructions. The transfection medium was changed 8 hours after transfection, and the lentiviral particles were collected 24 and 48 hours after transfection. Viral supernatant was used with 10 μg/mL polybrene (TR-1003-G, Sigma-Aldrich) to infect the cells, which were seeded at a confluence of 50% the day prior to transduction. Cells were incubated overnight with the viral supernatant, washed with PBS, and then supplemented with complete medium. Antibiotic selection (puromycin 2 μg/mL) was performed at least 72 hours after infection. The *Postn*-overexpressing (OE) lentiviral vector was purchased from VectorBuilder.

### RNA extraction, PCR, and qRT-PCR

Total RNA was extracted from *in vitro* and *ex vivo* FACS-sorted cells using TRIzol (Invitrogen) according to the manufacturer’s instructions. RNA was directly reverse-transcribed using the High-Capacity cDNA Reverse Transcription Kit (Applied Biosystems) according to the manufacturer’s instructions. Five to ten nanograms of RNA were used for each PCR. qPCRs were carried out using Power SYBR Green PCR Master Mix (Applied Biosystems) and the QuantStudio 3 real-time PCR system (Applied Biosystems). Primers used in qRT-PCR are listed in Supplementary Table S1.

### Flow cytometry and FACS-sorting

Cells were analyzed using the ID7000 Spectral Cell Analyzer (SONY), BD FACSymphony (BD), and CytoFLEX LX (Beckman). Cells were sorted using the FACSAria III (BD, Pharmingen). The following antibodies (BioLegend) were used to identify immune cells: anti-CD45 FITC, anti-CD31 FITC, anti-Ter119 FITC, anti-CD8 FITC, anti-CD45 Pacific Blue, anti-CD4 PE, anti-CD11b APC, anti-Ly6C APC-Cy7, anti-F4/80 FITC, and PE (Supplementary Table S2). Tumors were digested to a single-cell suspension enzymatically and filtered twice through 70 μm filters. Red blood cells were lysed with ACK solution (Gibco), washed twice with Cell Staining Buffer (BioLegend), and then stained with the fluorophore-conjugated antibodies for 30 minutes at 4°C. The excess unbound antibodies were washed out before acquisition in flow cytometry. For FACS-sorting experiments, the samples were prepared as for the flow cytometry analysis. Sorted cells were pelleted by centrifugation and lysed in TRIzol for RNA extraction right after sorting.

### Western blot

Protein lysates were prepared with ice-cold RIPA buffer (Boston BioProducts) supplemented with protease and phosphatase inhibitors (Roche). Protein lysates were separated using SDS-PAGE (precast gels, Thermo Fisher Scientific) and transferred to a nitrocellulose membrane. After blocking the membrane with 5% milk in PBS with 0.1% Tween-20 (PBST; Sigma), the membranes were incubated overnight at 4°C with anti-POSTN, anti-AKT, p-AKT, ERK-42/44, or p-ERK-42/44 (Cell Signaling Technology) diluted in PBST with 5% BSA (Supplementary Table S2). The blots were washed three times and then incubated with secondary antibodies (anti-Rabbit HRP, Thermo Fisher Scientific) diluted in PBST with 5% milk. Finally, the membranes were incubated with enhanced chemiluminescence (ECL) substrate (Pierce) for 1 minute and exposed for signal detection with the iBright Imager (Thermo Fisher Scientific).

### ELISA array

To detect soluble proteins, p53^KO^Ccne1^OE^ and p53^KO^Vgll3^OE^ tumor masses were collected and mechanically dissociated in 2 mL of PBS. Cells and debris were removed by centrifugation. The concentration of soluble protein in the supernatant was quantified with the Bradford assay (Bio-Rad) and analyzed using the Proteome Profiler Mouse XL Cytokine Array (ARY028, R&D Systems), following the manufacturer’s instructions. For the analysis of POSTN levels, the DY2955 kit was used (R&D Systems).

### Generation of bone marrow-derived macrophages (BMDM) and cell migration assay

To derive monocytes, long bones (tibia and femur) were collected from C57BL/6 mice and crushed. The cell suspension was first treated with ACK to eliminate red blood cells, and after two washes in PBS, it was seeded at a concentration of 2 million cells/mL in DMEM with 10% FBS, supplemented with 50 ng/mL M-CSF. The medium was replaced with fresh medium every other day. Cells at different time points were collected for Transwell assays or treatment with recombinant POSTN (2955-F2-050, R&D Systems). Recombinant mouse POSTN or the conditioned media collected from sarcoma cells *Postn*-OE was added to the bottom chamber with complete cell media (DMEM, 10% FBS, 1% penicillin–streptomycin) in a 24-well plate containing polystyrene Transwell membranes with 3 μm pores. One million BMDM cells were seeded in the upper chamber and allowed to migrate through the chamber pores overnight. The Transwell inserts were then fixed in 10% formalin, and the cells were stained with crystal violet. Any remaining cells in the top chamber were physically removed, and only the cells that passes through were quantified by solubilizing the crystal violet in 10% acetic acid.

### Anchorage-independent cell growth (soft-agar assays)

Soft agar colony formation assay was carried out by seeding 1 × 10^4^ sarcoma cells in DMEM containing 0.3% low-melting agarose and 10% FBS. The cells were then plated in six-well plates previously coated with DMEM containing 0.6% low-melting agarose and 10% FBS. The number of colonies was scored 2 weeks later, and quantification was completed using ImageJ. Briefly, at least 10 pictures were taken from each experimental condition. The images were processed through ImageJ, in which a common threshold of detection was applied to all the pictures. Based on this, the system determined the area covered by the colonies, considering both the number of colonies and their areas. Representative pictures are shown in Fig. 2B and Supplementary Fig. 2C.

### Statistical analysis

No statistical method was used to predetermine sample size. For all statistical analyses, the analysis was done using a two-tailed unpaired Student *t* test. Values of *P* < 0.05 were considered statistically significant. *, *P* < 0.05; **, *P* < 0.01; ***, *P* < 0.001 (*t* test).

## Results

### POSTN promotes sarcoma growth *in vivo*

**Figure 1.**
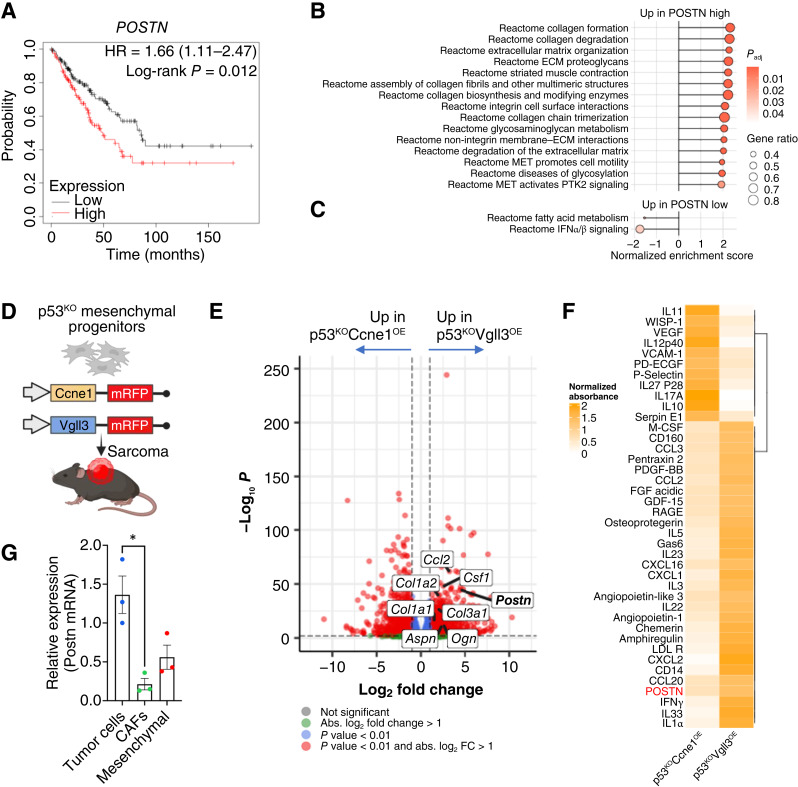
*POSTN* expression is correlated with patient survival and expression of extracellular matrix proteins. **A,** Kaplan–Meier plot showing reduced survival for patients with sarcoma expressing high *POSTN* levels. **B** and **C,** Pathway analysis of patients with sarcoma showing high vs. low POSTN expression levels revealed increased expression of ECM-related elements in POSTN-high patients. **D,** Schematic representation of the sarcoma mouse models. **E,** Volcano plot of select differentially expressed genes in tumor cells sorted from the p53^KO^Vgll3^OE^ (positive log fold change) vs. p53^KO^Ccne1^OE^ (negative log fold change) mouse tumors. **F,** Heat map showing the most differentially expressed soluble factors in the ELISA array comparing p53^KO^Vgll3^OE^ vs. p53^KO^Ccne1^OE^ whole tumors. **G,** Expression of *Postn* mRNA in tumor cells, adjacent CAFs, and bone marrow–derived mesenchymal cells (CD45^−^ PDGFRA^+^). Unless otherwise indicated, results are presented as mean ± SEM, and *P* values were derived from a two-tailed unpaired Student *t* test (*, *P* < 0.05). [Created in BioRender. Guarnerio, J. (2025) https://BioRender.com/fo00cpj.]

Due to their mesenchymal origin, STS cells produce and deposit large amounts of ECM components (10). A high ECM-related gene expression score (“matrix index”) has been associated with worse overall survival in the TCGA cohort of patients with sarcoma (10, 29). However, the functional roles of individual ECM components in STS remain to be elucidated. Recent studies have shown that elevated levels of POSTN, detected in tumor tissue by IHC as well as in peripheral blood, correlate with poor prognosis in patients with STS (26). Although POSTN has also been linked to poor outcomes in epithelial cancers—primarily due to its role in promoting epithelial-to-mesenchymal transition (EMT) and metastasis (30–33)—its functional role in STS has not been thoroughly investigated. By analyzing TCGA patient data, we confirmed that high *POSTN* mRNA expression correlates with poor prognosis in sarcoma, similar to trends seen in several carcinomas (Fig. 1A; Supplementary Fig. S1A). Furthermore, *POSTN* expression positively correlates with the expression of other ECM-related genes, including collagens (Fig. 1B and C) and various proteoglycans [e.g., decorin (*DCN*), asporin (*ASPN*), and lumican (*LUM*); Supplementary Fig. S1B]. Interestingly, *POSTN* expression also showed modest correlation with myeloid cell markers such as *CD14* (expressed by bone marrow–derived monocytes), *CSF1R* (commonly marking tumor–recruited monocytes), and *CD163* (a marker of TAMs; Supplementary Fig. S1C). These associations suggest a potential link between high POSTN expression and increased myeloid cell infiltration into the tumor.

To functionally explore the role of POSTN in sarcoma, we examined its expression in two genetically engineered mouse models of undifferentiated pleomorphic sarcoma (UPS) developed in our lab, each of which mimics common genomic alterations observed in patients with UPS (34): p53^KO^Ccne1^OE^ and p53^KO^Vgll3^OE^ (Fig. 1D). Analysis of *ex vivo* mRFP+ sorted tumor cells revealed significantly higher levels of *Postn* expression in p53^KO^Vgll3^OE^ tumors compared with p53^KO^Ccne1^OE^ tumors (Fig. 1E; Supplementary Fig. S1D). As in patient samples, higher *Postn* expression in p53^KO^Vgll3^OE^ tumor cells was associated with elevated levels of other ECM-related genes [e.g., *Col1a1*, osteoglycin (*Ogn*), and *Aspn*] and cytokines known to recruit monocytes, such as *Csf1* and *Ccl2* (Fig. 1E). These findings were confirmed at the protein level using ELISA arrays, which showed higher concentrations of secreted CCL2, CSF1, and POSTN in the p53^KO^Vgll3^OE^ tumor milieu compared with p53^KO^Ccne1^OE^ tumors (Fig. 1F). Moreover, we found that sarcoma cells expressed higher levels of *Postn* compared with cancer-associated fibroblasts (CAF) isolated from the same tumor mass and normal mesenchymal cells isolated from bone marrow (Fig. 1G), suggesting that tumor cells are the main producers of *Postn* in the sarcoma mass. However, although both mouse models expressed *Postn in vivo*, this expression was lost *in vitro*, suggesting that *Postn* may be regulated by signals from the TME (Supplementary Fig. S1E).

**Figure 2.**
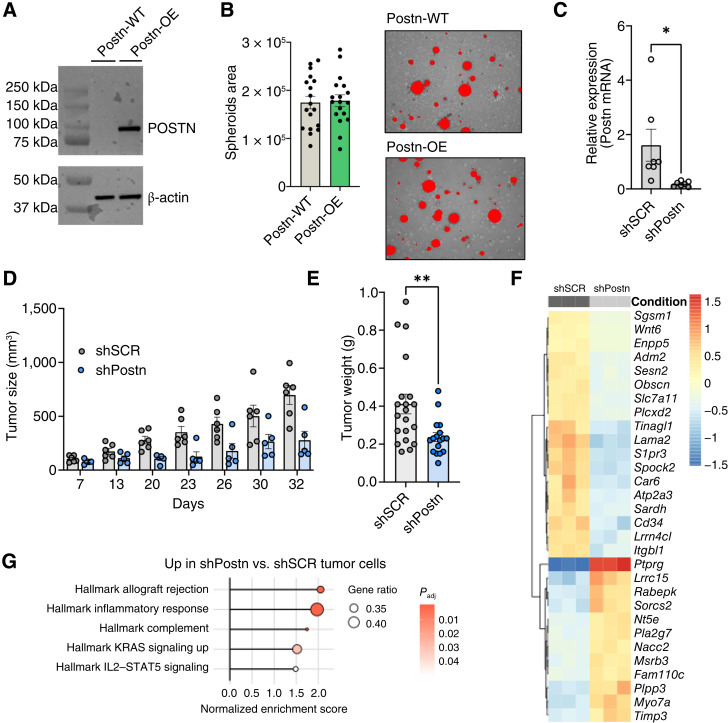
*Postn* silencing reduces tumor growth in the p53^KO^Vgll3^OE^ mouse model. **A,** Western blot analysis of POSTN expression in murine Postn-WT and Postn-OE sarcoma cells (p53^KO^Vgll3^OE^). **B,** Soft agar assays comparing Postn-WT and Postn-OE sarcoma cells (p53^KO^Vgll3^OE^). Representative images of the analyzed spheroids (right) and spheroid area quantification (left) are shown. Each dot represents a quantified imaging area. **C,** Relative expression of Postn in FACS-sorted tumor cells upon Postn silencing through shRNAs (shPostn) in p53^KO^Vgll3^OE^ sarcoma cells. **D,** Tumor size at different time points, comparing Postn-WT (shSCR) and Postn-silenced (shPostn) sarcoma cells (p53^KO^Vgll3^OE^). **E,** Weight of the tumor masses at the experimental endpoint, comparing tumors generated by Postn-WT (shSCR) and Postn-silenced (shPostn) sarcoma cells (p53^KO^Vgll3^OE^). **F,** Heatmap of the top differentially expressed genes with absolute log_2_ fold change > 1. **G,** Top pathways enriched in shPostn vs. shSCR sarcoma cells (p53^KO^Vgll3^OE^). No pathways were enriched in shSCR vs. shPostn tumor cells. Unless otherwise indicated, results are presented as mean ± SEM, and *P* values were derived from a two-tailed unpaired Student *t* test (*, *P* < 0.05; **, *P* < 0.01). All experiments were performed in the p53^KO^Vgll3^OE^ sarcoma model. WT, wild type.

We conducted *in vitro* experiments to examine POSTN’s functional role. Overexpression of *Postn* via viral vectors in p53^KO^Vgll3^OE^ sarcoma cells was successful in eliciting POSTN protein expression (Fig. 2A), but this did not affect proliferation in either 2D cultures or 3D soft agar spheroids, even under nutrient-deprived conditions (2% FBS; Fig. 2B; Supplementary Fig. S2A). Similar results were observed in p53^KO^Ccne1^OE^ sarcoma cells (Supplementary Fig. S2B and S2C). Moreover, treatment of human sarcoma cells with recombinant POSTN did not alter the activation of AKT or MAPK pathways (Supplementary Fig. S2D)—pathways previously implicated in POSTN signaling in other cancer types (33, 35)—suggesting that in sarcoma, *Postn* regulation of tumor growth may not function primarily through tumor cell–intrinsic mechanisms.

Because the p53^KO^Ccne1^OE^ tumor cells exhibited lower endogenous *Postn* expression than the p53^KO^Vgll3^OE^ model, further experiments focused on the latter model (p53^KO^Vgll3^OE^). Accordingly, to further investigate the functional role of POSTN *in vivo*, we silenced *Postn* expression in the p53^KO^Vgll3^OE^ UPS cells using shRNA and validated silencing via qRT-PCR on FACS-sorted *ex vivo* tumor cells (Fig. 2C). Contrary to *in vitro* observations, tumors with *Postn* knockdown showed reduced growth rates and lower tumor weights compared with control tumors (Fig. 2D and E), suggesting that POSTN might affect tumor growth through non-tumor cell–intrinsic mechanisms. To investigate the mechanisms underlying *Postn*’s impact on tumor growth, we next performed RNA sequencing (RNA-seq) on FACS-sorted *Postn*-silenced and control tumor cells (Fig. 2F) and compared gene expression in the two conditions. The top upregulated gene in the *Postn*-silenced tumor cells was the protein tyrosine phosphatase receptor type G (*Ptprg*), a tumor suppressor gene that plays critical roles in different tumor types, including sarcoma (36), by inhibiting tumor cell invasion and tumor angiogenesis through affecting the Akt signaling pathway (37, 38). Interestingly, no hallmark gene sets related to the cell cycle or proliferation were significantly different in the *Postn*-silenced cells despite the apparently faster growth of those tumors. This accorded with the earlier finding that *in vitro*, there was no effect of *Postn* on proliferation or spheroid formation (Fig. 2B; Supplementary Fig. S2A–S2C). Instead, GSEA revealed that the top gene sets upregulated in *Postn*-silenced samples were those related to the inflammatory response; four of the five significantly upregulated gene sets pertained to immune signaling—for example, Hallmark inflammatory response and Hallmark IL2/STAT5 signaling (Fig. 2G)—with only one enriched gene set related to a known tumor hallmark (KRAS signaling). This result suggested that *Postn* silencing had minimal effects on tumor cell–intrinsic gene expression—which may explain the lack of significant growth difference in the *in vitro* assays—but that *Postn* may exert influence via the microenvironment and thus impinge on tumor growth only *in vivo*, in the full context of the immune compartment.

### POSTN suppression is associated with the reshaping of the tumor immune microenvironment

**Figure 3.**
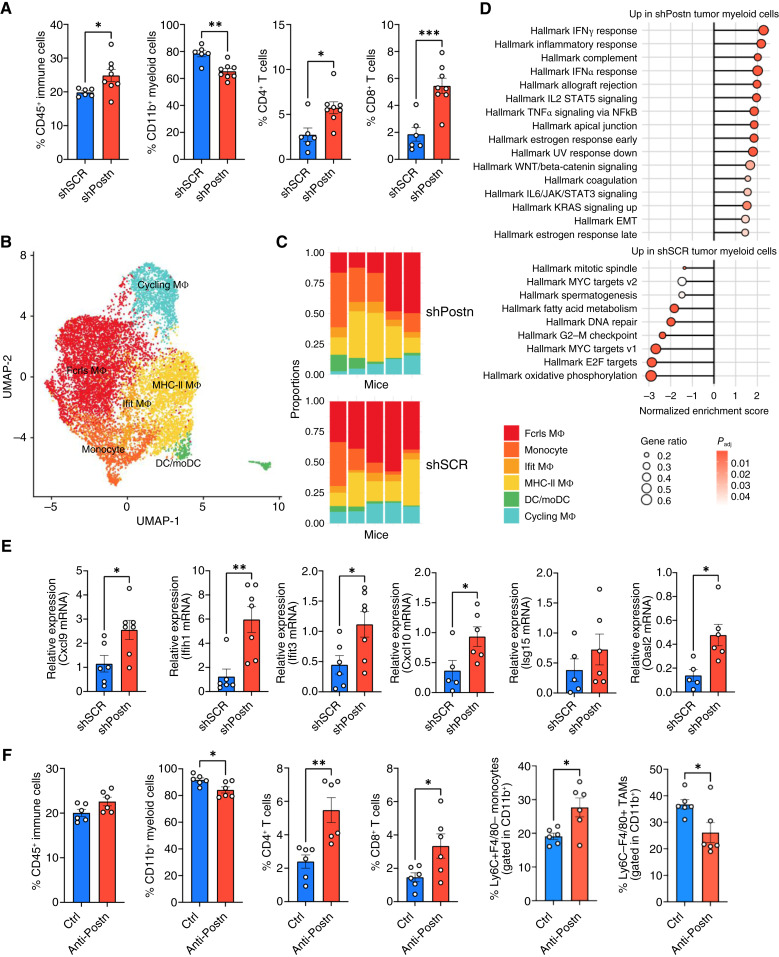
*Postn* silencing in tumor cells shapes the sarcoma immune microenvironment. **A,** Flow cytometry analysis of CD45^+^ immune cells and different types of immune cells within the CD45^+^ population in the microenvironment of Postn-WT (shSCR) and Postn-silenced (shPostn) tumors (p53^KO^Vgll3^OE^). Each dot represents one mouse. **B,** Subclustering of the mononuclear phagocyte compartment (macrophages, monocytes, and dendritic cells) from scRNA-seq. **C,** Proportions of the subclusters from **B** in individual shSCR or shPostn tumors (*n* = 5 mice in each group). **D,** Pathways enriched in mononuclear phagocytes from shPostn tumors (top) or from shSCR tumors (bottom). **E,** Relative expression of IFN-stimulated and proinflammatory genes in CD11b^+^ myeloid cells FACS-sorted from mouse tumors, generated by either *Postn*-WT (shSCR) or *Postn*-silenced (shPostn) sarcoma cells. Each dot represents one mouse. **F,** Flow cytometry analysis of CD45^+^ immune cells and different types of immune cells within the CD45^+^ population in the microenvironment of mice treated with POSTN-blocking antibody (anti-POSTN). Each dot represents one mouse. Unless otherwise indicated, results are presented as mean ± SEM, and *P* values were derived from a two-tailed unpaired Student *t* test (*, *P* < 0.05; **, *P* < 0.01; ***, *P* < 0.001). Experiments were performed in the p53^KO^Vgll3^OE^ model.

Observing minimal tumor cell–autonomous effects of *Postn*, we next investigated whether *Postn* expression by tumor cells *in vivo* could influence the immune composition of the TME. To this end, we implanted p53^KO^Vgll3^OE^ tumor cells [either *Postn* wild-type (shSCR) or *Postn*-silenced (shPostn)] into syngeneic, immunocompetent mice. Tumors were harvested at matched time points, and their immune cell composition was analyzed by flow cytometry. Interestingly, tumors lacking *Postn* expression showed an overall increase in infiltrating CD45^+^ immune cells (Fig. 3A). Within this population, we observed a decrease in CD11b^+^ myeloid cells alongside increased proportions of both CD4^+^ and CD8^+^ T cells (Fig. 3A). Given the earlier observed correlation between high *POSTN* expression and myeloid-related gene signatures in patients with sarcoma (Fig. 1C), we further investigated whether and how *Postn* influences myeloid cells in the tumor. Accordingly, we first performed single-cell RNA-seq (scRNA-seq) to comprehensively characterize the impact of *Postn* on myeloid cell states in the TME.

As shown in Fig. 3B, six distinct myeloid subclusters were identified (see also Supplementary Fig. S3A), including *Fcrls*^+^ TAMs, IFN-responsive macrophages, and MHC-II^+^ antigen-presenting macrophages, as well as monocytes and monocyte-derived dendritic cells (moDCs). Using sample-specific hashtags to trace cells from individual mice (Fig. 3C; Supplementary Fig. S3B), we compared the relative abundance of these subclusters across conditions. Notably, monocytes and IFN-responsive macrophages were slightly enriched in *Postn*-silenced tumors relative to controls, whereas on the contrary, *Fcrls*-TAMs slightly decreased (Fig. 3C). Considering the entire myeloid population from each mouse as pseudobulk transcriptomes, we found that IFN-related pathways were enriched in the myeloid cells populating *Postn*-silenced tumors compared with the controls (Fig. 3D). As IFN signaling and proinflammatory myeloid populations play key roles in constraining tumor progression, we validated these scRNA-seq findings with additional experiments. CD11b^+^ myeloid cells were FACS-sorted from *Postn* wild-type and *Postn*-silenced tumors and analyzed via qRT-PCR. As shown in Fig. 3E, several proinflammatory genes were upregulated in myeloid cells from *Postn*-silenced tumors, supporting the transcriptional shifts observed in scRNA-seq.

To further corroborate these observations, we performed a complementary experiment in which *Postn* expression was enhanced in the p53^KO^Ccne1^OE^ sarcoma model. Accordingly, we found that overexpression of *Postn* favored tumor growth (Supplementary Fig. S3C), as well as the presence of macrophages (TAM) in the tumor mass (Supplementary Fig. S3D).

Finally, to assess whether POSTN targeting could have therapeutic potential, we treated tumor-bearing mice with a neutralizing anti-POSTN antibody. Although antibody treatment alone did not significantly reduce tumor growth (Supplementary Fig. S3E), it did alter the immune composition of the TME. Specifically, anti-POSTN treatment reduced TAM proportions and increased monocytes, as well as CD4^+^ and CD8^+^ T cells—key players in antitumor immunity (Fig. 3F).

### POSTN recruits myeloid cells to the tumor mass

**Figure 4.**
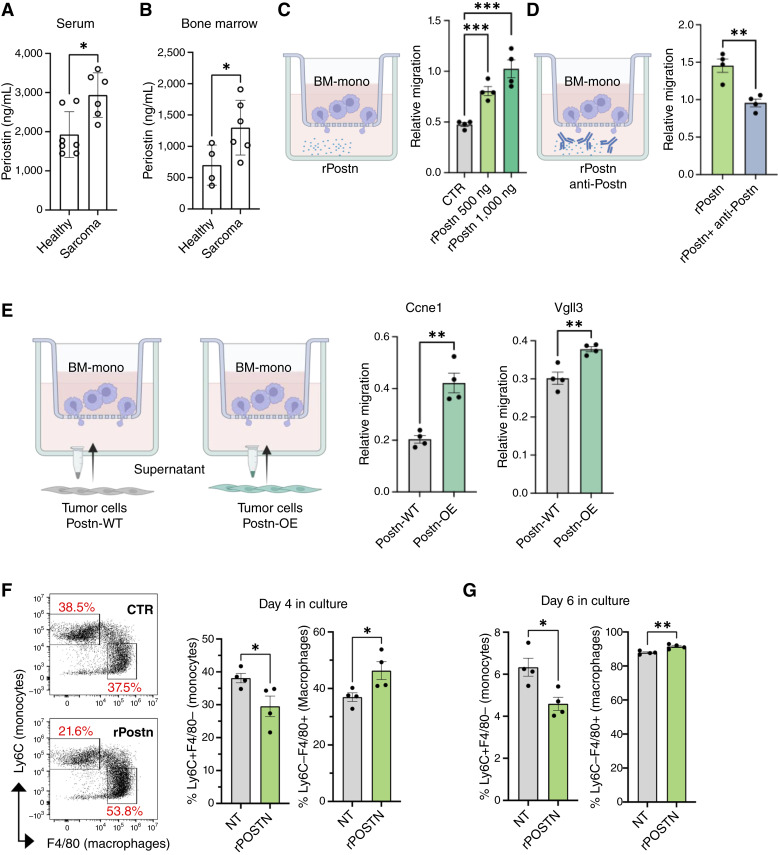
*Postn* recruits bone marrow monocytes and promotes the accumulation of macrophages. **A,** Quantification of POSTN levels in the serum of sarcoma-bearing mice compared with healthy mice. Each dot represents one mouse. **B,** Quantification of POSTN levels in the bone marrow of sarcoma-bearing mice compared with healthy mice. Each dot represents one mouse. **C,** Transwell migration assays to measure the capability of bone marrow–derived monocytes to migrate toward recombinant POSTN protein after 16 hours of exposure or (**D**) in the presence of POSTN-neutralizing antibodies. **E,** Transwell migration assays to assess the capability of bone marrow–derived monocytes to migrate toward supernatant from sarcoma cells expressing wild-type (Postn-WT) or enhanced *Postn* levels (Postn-OE). **F** and **G,** Analysis of monocytes (Ly6C+F4/80^−^ cells) and macrophages (Ly6C-F4/80^+^ cells) at two different time points (days 4 and 6) in culture with recombinant POSTN protein. Unless otherwise indicated, results are presented as mean ± SEM, and *P* values are derived from a two-tailed unpaired Student *t* test (*, *P* < 0.05; **, *P* < 0.01; ***, *P* < 0.001). For **C–G**, each dot represents BMDM derived from a single mouse (*n* = 4 independent mice). [Created in BioRender. Guarnerio, J. (2025) https://BioRender.com/fo00cpj.]

Recent studies have reported a correlation between elevated POSTN levels in the blood and poor prognosis in patients (26). To evaluate whether POSTN is similarly elevated in our sarcoma mouse model, we collected plasma samples and measured POSTN levels using ELISA. Consistent with patient data, we detected elevated levels of POSTN in the circulation of tumor-bearing mice (Fig. 4A), as well as in their bone marrow (Fig. 4B)—a site in which POSTN is known to influence bone homeostasis and the hematopoietic stem cell niche (39). Given that POSTN secreted by tumor cells reaches the bone marrow, we hypothesized that it may act as a chemoattractant, recruiting myeloid cells from the bone marrow to the tumor site. To test this, we conducted *in vitro* Transwell migration assays, placing bone marrow–derived monocytes in the upper chamber and recombinant POSTN in the lower chamber. We observed a dose-dependent increase in monocyte migration toward POSTN (Fig. 4C), which was reduced in the presence of POSTN-neutralizing antibodies (Fig. 4D), supporting the idea that POSTN functions as a chemoattractant for bone marrow–derived monocytes. To further validate this, we used conditioned media from tumor cells overexpressing *Postn* (Postn-OE) or from wild-type tumor cells (Postn-WT, which typically downregulate *Postn in vitro*, Supplementary Fig. S1E). Conditioned medium from cells engineered to overexpress *Postn* stimulated increased monocyte migration compared with the wild-type *Postn* control (Fig. 4E), reinforcing the chemoattractant role of POSTN.

Next, we explored whether POSTN also influences monocyte-to-macrophage maturation. *In vitro* differentiation assays showed that recombinant POSTN decreased the proportion of cells retaining monocyte markers and increased the expression of macrophage-associated markers (Fig. 4F–G), suggesting that POSTN not only attracts monocytes but also promotes their maturation once they enter the TME. However, when we assessed the expression of classical secreted factors for macrophages, we found no significant differences between control and recombinant POSTN-treated macrophages *in vitro* (Supplementary Fig. S4). This suggests that POSTN may not directly influence the macrophage secretome or, alternatively, that *in vitro* systems may not fully recapitulate the complexity of macrophage activation states observed *in vivo*.

## Discussion

Our study identifies POSTN as a key ECM component expressed by sarcoma cells *in vivo*, with a previously underappreciated role in immune remodeling and sarcoma progression. Although POSTN has been extensively studied in epithelial malignancies—in which it is typically upregulated during EMT—its function in mesenchymal tumors such as STS has remained largely unexplored. In carcinomas, POSTN is expressed by tumor cells undergoing EMT and is associated with increased metastatic potential (33), maintenance of cancer stem cell populations (40), and resistance to chemotherapy and radiation (41–43). These effects are mediated, at least in part, through the activation of canonical oncogenic signaling pathways, including PI3K/AKT, Wnt/β-catenin, and MAPK/ERK (31, 33, 44). For example, in breast and colon cancers, POSTN expression supports cancer stemness and correlates with poor clinical outcomes (33, 45, 46), reinforcing its importance in tumor aggressiveness and therapeutic resistance.

In epithelial tumors, the TME plays a critical role in POSTN regulation. CAFs—the dominant nonimmune stromal cells in carcinomas—are the main source of ECM proteins, including POSTN (47, 48). CAF-derived POSTN has been shown to promote tumor growth, therapy resistance, and stem cell maintenance through paracrine signaling. Importantly, POSTN may also facilitate immune evasion by modulating chemokine gradients, enhancing matrix stiffness, and contributing to the physical and biochemical exclusion of cytotoxic T cells from the tumor parenchyma.

Interestingly, in sarcomas—which are of mesenchymal origin—tumor cells themselves serve as the primary source of POSTN. This distinction led us to initially hypothesize that POSTN could act through tumor cell–intrinsic mechanisms, similar to those observed in carcinomas. However, our data demonstrated that POSTN had minimal impact on tumor cell–autonomous signaling pathways or proliferative capacity, even when directly manipulated *in vitro* or delivered exogenously as recombinant protein. These findings indicate that the tumor-promoting functions of POSTN in sarcoma are primarily exerted through noncell-autonomous mechanisms.

Our investigation into the TME revealed that POSTN plays a critical role in shaping immune cell composition. Studies in different tumor types have reported that high POSTN expression correlates with increased macrophage infiltration and immunosuppressive polarization, leading to worse clinical outcomes (49, 50). Outside of cancer, POSTN has been implicated in the recruitment of bone marrow–derived monocytes in models of neuroinflammation and tissue injury (51).

Consistent with these findings, we observed that tumor-derived POSTN in sarcoma acts as a chemoattractant for monocytes and promotes their differentiation into TAMs. These effects were supported by *in vitro* migration assays using recombinant POSTN and conditioned media from POSTN-expressing tumor cells, as well as *in vivo* profiling by scRNA-seq. Importantly, we found that POSTN-silenced tumors exhibited increased infiltration of CD4^+^ and CD8^+^ T cells, along with a reduction in immunosuppressive myeloid subsets, including TAMs. This myeloid remodeling is particularly relevant in sarcomas, which are typically considered “immune-cold” tumors with low T-cell infiltration and a heavy reliance on myeloid-derived suppressor cells and TAMs for maintaining an immunosuppressive niche.

These findings suggest that modulating the POSTN–myeloid cell axis may offer a promising therapeutic strategy. Given that POSTN targeting alone was insufficient to induce tumor regression in our models, we propose that POSTN inhibition could be best deployed as part of combinatorial strategies—either with immune checkpoint inhibitors (e.g., anti–PD-1/PD-L1; ref. 52), agents that enhance T-cell function, or novel modalities such as fibrosis-targeting chimeric antigen receptor (CAR) T cells that degrade ECM and improve immune infiltration (53, 54). The reprogramming of TAMs toward a proinflammatory phenotype in response to POSTN depletion may further sensitize tumors to T cell–mediated cytotoxicity, enhancing the efficacy of such therapies.

Several limitations of this study should be acknowledged. First, our conclusions are based on genetically engineered mouse models of sarcoma that better recapitulate undifferentiated sarcoma. Given the heterogeneity of STS, future studies across multiple subtypes and models are necessary to determine the broader applicability of our findings or whether POSTN plays a role only in undifferentiated sarcoma in patients. Additionally, as only female mice were used in this work, possible sex-specific effects and antitumor immune responses may be further assessed. Second, although we observed remodeling of the immune microenvironment upon POSTN inhibition, we did not evaluate the effects of combining POSTN targeting with standard-of-care chemotherapy or immunotherapeutic agents. Such combinatorial approaches could offer valuable insights into the clinical relevance and potential for translational application.

Third, although our data support a role for POSTN in immune microenvironment modulation, we did not dissect possible autocrine mechanisms and the underlying mechanisms by which POSTN reshapes immune cell composition and function. One possibility is that POSTN acts directly on TAMs via integrin-mediated signaling. Alternatively, POSTN may exert indirect effects through other pathways—for instance, by suppressing PTPRG, which we identified as one of the most upregulated genes in POSTN-silenced tumors. We may speculate that PTPRG acts similarly to another member of the family, PTPRO, which regulates proinflammatory and anti-inflammatory/proregenerative macrophages in cancer (55). These hypotheses warrant further mechanistic investigation.

Lastly, although POSTN is known to regulate bone remodeling and the hematopoietic stem cell niche (56), we did not assess how tumor-derived systemic POSTN might affect myelopoiesis or immune cell development via alterations in the bone marrow microenvironment. Addressing these questions would require dedicated *in vivo* studies to evaluate the broader systemic effects of POSTN on hematopoietic and immune function. At the same time, experiments focused on measuring a possible effect of Postn on tissue-resident macrophages, in addition to the recruited ones, may be informative.

In summary, our work reveals POSTN as a tumor-derived ECM factor that orchestrates immunosuppressive remodeling of the sarcoma microenvironment via regulation of the myeloid compartment. These insights may open new avenues for targeting stromal–tumor–immune interactions in sarcoma and underscore the importance of considering ECM-derived cues as therapeutic vulnerabilities in mesenchymal malignancies.

## Supplementary Material

Supplementary Figure S1 Figure S1. POSTN expression is correlated with patient survival and expression of macrophage-associated genes.

Supplementary Figure S2 Figure S2. Postn does not regulate tumor cell growth in vitro.

Supplementary Figure S3 Figure S3. Postn silencing shapes the immune sarcoma microenvironment.

Supplementary Figure S4 Figure S4. Differential expression of macrophage-related genes in BMDM vs BMDM cultured with recombinant POSTN (rPOSTN).

Supplementary Table 1 Ab table

Supplementary Table 2 primers table

## Data Availability

The raw RNA-seq and scRNA-seq data in FASTQ format were deposited in the NCBI Sequence Read Archive (accession number: GSE302846). Other data generated in this study are available from the corresponding author upon request.
