# Supplementary material for: Periostin Promotes Sarcoma Growth by Promoting Tumor-Associated Macrophage Migration and Differentiation
Source: Cancer Res Commun. 2025 Dec 26;5(12):2224–35. doi: 10.1158/2767-9764.CRC-25-0301 (PMC12740715; doi:10.1158/2767-9764.CRC-25-0301)
Supplement: Supplementary Figure S1 — Figure S1. POSTN expression is correlated with patient survival and expression of macrophage-associated genes. [file crc-25-0301_supplementary_figure_s1_suppsf1.pptx]

## Slide 1
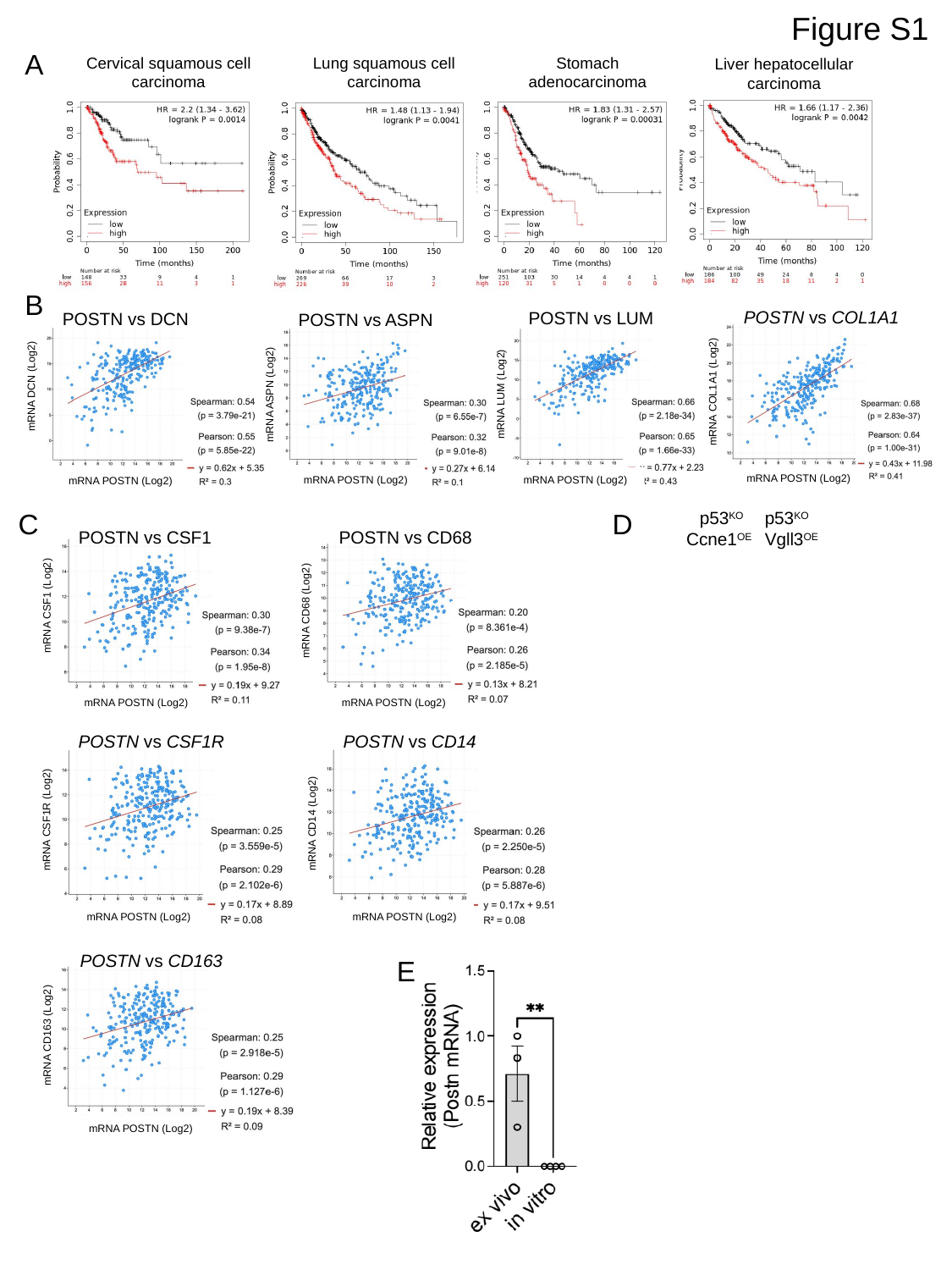

Figure S1
A
Cervical squamous cell carcinoma
Stomach adenocarcinoma
Lung squamous cell carcinoma
Liver hepatocellular carcinoma
B
POSTN vs COL1A1
POSTN vs LUM
POSTN vs DCN
POSTN vs ASPN
mRNA DCN (Log2)
mRNA COL1A1 (Log2)
mRNA LUM (Log2)
mRNA ASPN (Log2)
mRNA POSTN (Log2)
mRNA POSTN (Log2)
mRNA POSTN (Log2)
mRNA POSTN (Log2)
C
D
p53KO
Ccne1OE
p53KO
Vgll3OE
POSTN vs CSF1
POSTN vs CD68
mRNA CSF1 (Log2)
mRNA CD68 (Log2)
mRNA POSTN (Log2)
mRNA POSTN (Log2)
POSTN vs CSF1R
POSTN vs CD14
mRNA CSF1R (Log2)
mRNA CD14 (Log2)
mRNA POSTN (Log2)
mRNA POSTN (Log2)
POSTN vs CD163
E
mRNA CD163 (Log2)
mRNA POSTN (Log2)

## Slide 2
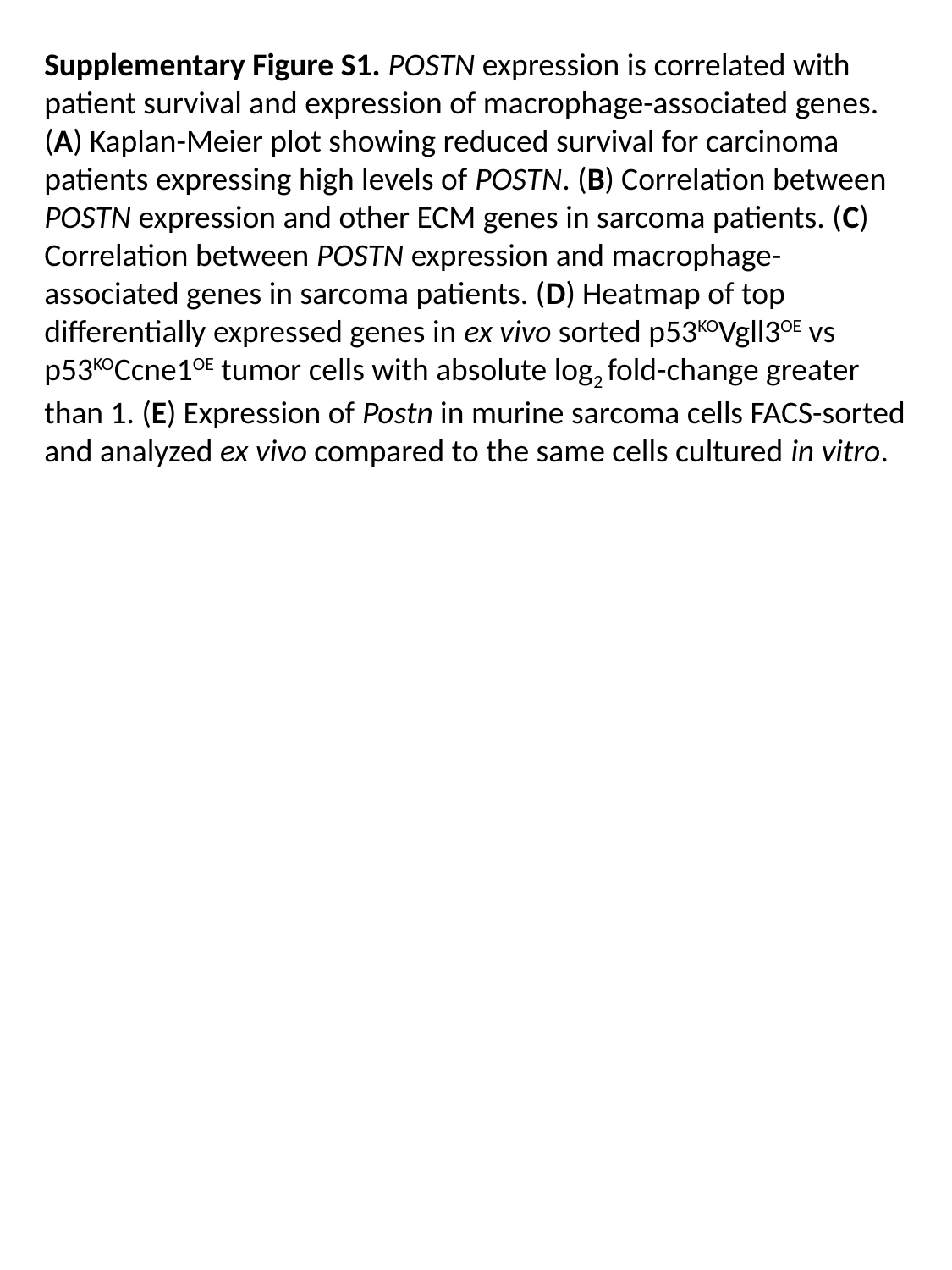

Supplementary Figure S1. POSTN expression is correlated with patient survival and expression of macrophage-associated genes. (A) Kaplan-Meier plot showing reduced survival for carcinoma patients expressing high levels of POSTN. (B) Correlation between POSTN expression and other ECM genes in sarcoma patients. (C) Correlation between POSTN expression and macrophage-associated genes in sarcoma patients. (D) Heatmap of top differentially expressed genes in ex vivo sorted p53KOVgll3OE vs p53KOCcne1OE tumor cells with absolute log2 fold-change greater than 1. (E) Expression of Postn in murine sarcoma cells FACS-sorted and analyzed ex vivo compared to the same cells cultured in vitro.
