# Supplementary material for: Periostin Promotes Sarcoma Growth by Promoting Tumor-Associated Macrophage Migration and Differentiation
Source: Cancer Res Commun. 2025 Dec 26;5(12):2224–35. doi: 10.1158/2767-9764.CRC-25-0301 (PMC12740715; doi:10.1158/2767-9764.CRC-25-0301)
Supplement: Supplementary Figure S2 — Figure S2. Postn does not regulate tumor cell growth in vitro. [file crc-25-0301_supplementary_figure_s2_suppsf2.pptx]

## Slide 1
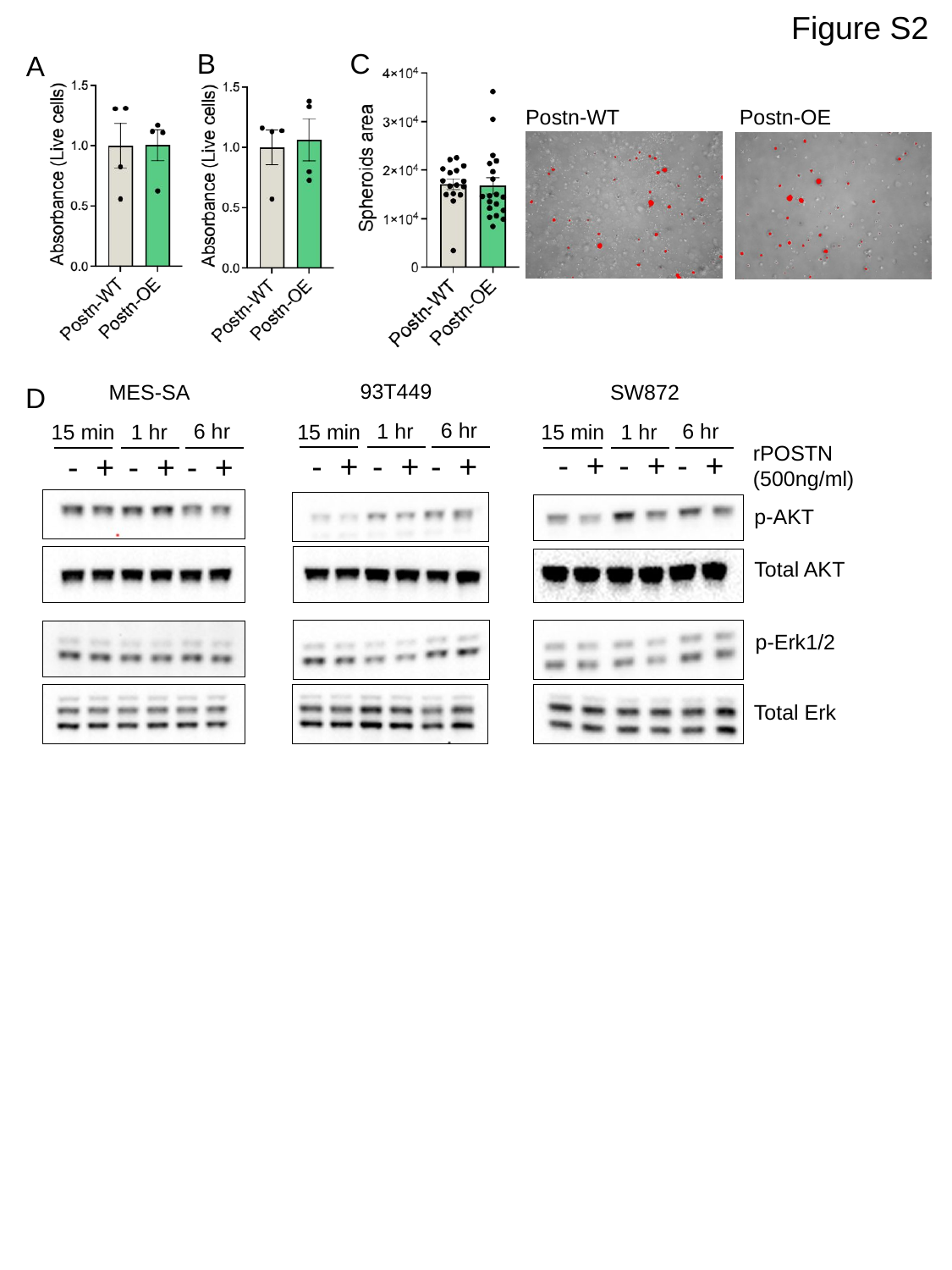

Figure S2
B
C
A
Postn-WT
Postn-OE
93T449
SW872
MES-SA
D
6 hr
1 hr
6 hr
6 hr
1 hr
1 hr
15 min
15 min
15 min
rPOSTN
(500ng/ml)
-
+
-
+
-
+
-
+
-
+
-
+
-
+
-
+
-
+
p-AKT
Total AKT
p-Erk1/2
Total Erk

## Slide 2
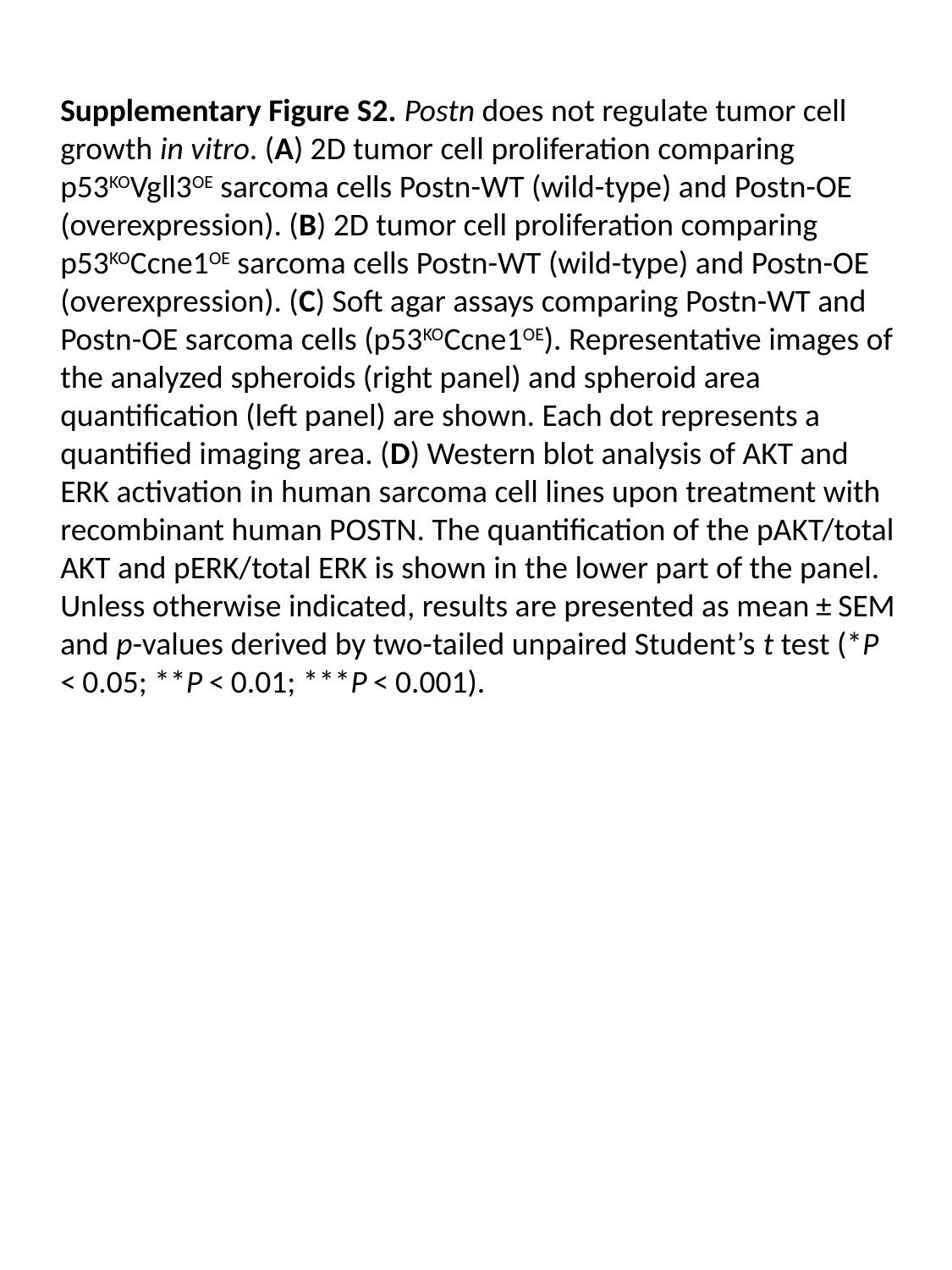

Supplementary Figure S2. Postn does not regulate tumor cell growth in vitro. (A) 2D tumor cell proliferation comparing p53KOVgll3OE sarcoma cells Postn-WT (wild-type) and Postn-OE (overexpression). (B) 2D tumor cell proliferation comparing p53KOCcne1OE sarcoma cells Postn-WT (wild-type) and Postn-OE (overexpression). (C) Soft agar assays comparing Postn-WT and Postn-OE sarcoma cells (p53KOCcne1OE). Representative images of the analyzed spheroids (right panel) and spheroid area quantification (left panel) are shown. Each dot represents a quantified imaging area. (D) Western blot analysis of AKT and ERK activation in human sarcoma cell lines upon treatment with recombinant human POSTN. The quantification of the pAKT/total AKT and pERK/total ERK is shown in the lower part of the panel. Unless otherwise indicated, results are presented as mean ± SEM and p-values derived by two-tailed unpaired Student’s t test (*P < 0.05; **P < 0.01; ***P < 0.001).
