# Supplementary material for: Periostin Promotes Sarcoma Growth by Promoting Tumor-Associated Macrophage Migration and Differentiation
Source: Cancer Res Commun. 2025 Dec 26;5(12):2224–35. doi: 10.1158/2767-9764.CRC-25-0301 (PMC12740715; doi:10.1158/2767-9764.CRC-25-0301)
Supplement: Supplementary Figure S3 — Figure S3. Postn silencing shapes the immune sarcoma microenvironment. [file crc-25-0301_supplementary_figure_s3_suppsf3.pptx]

## Slide 1
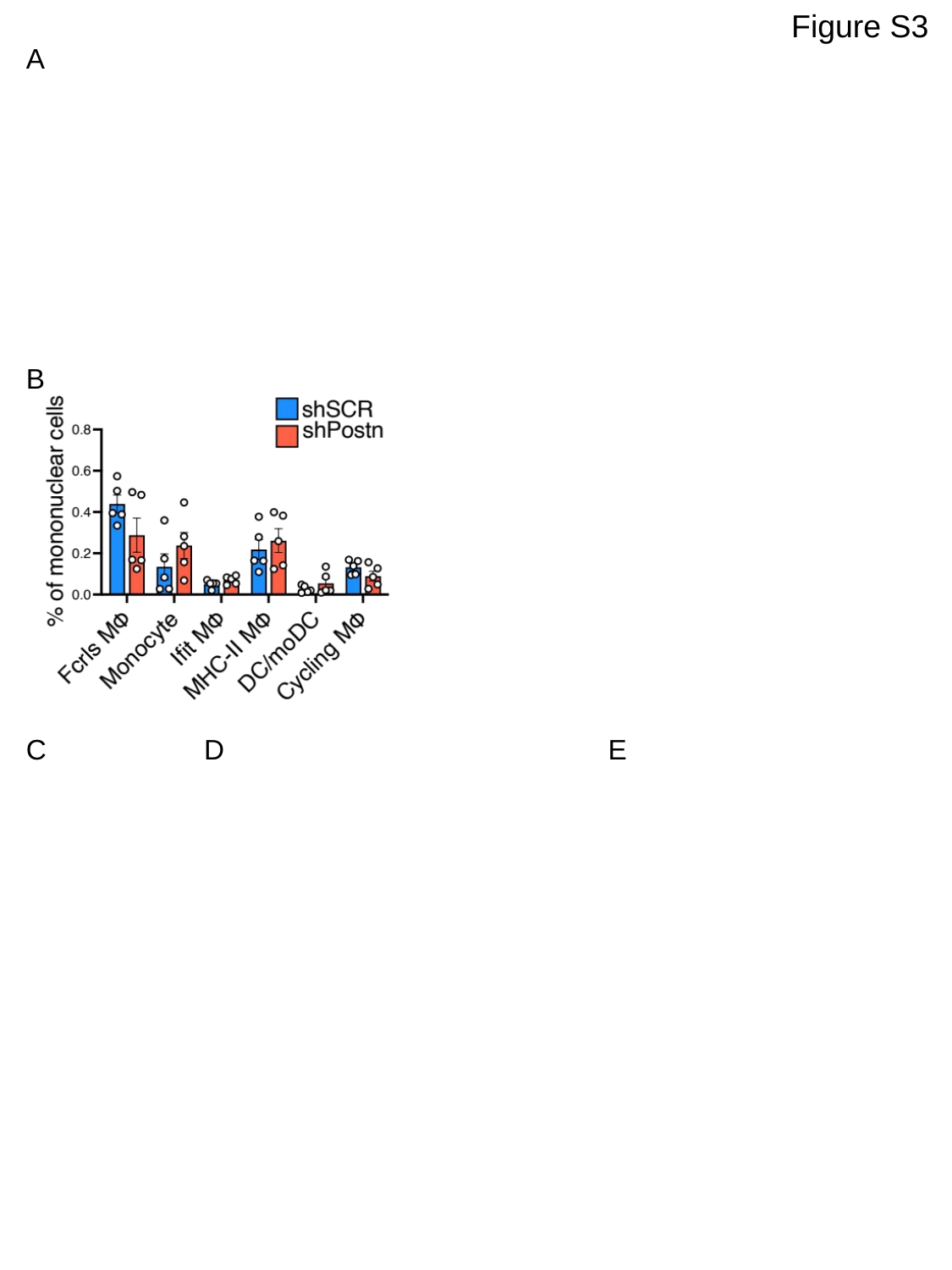

Figure S3
A
B
C
D
E

## Slide 2
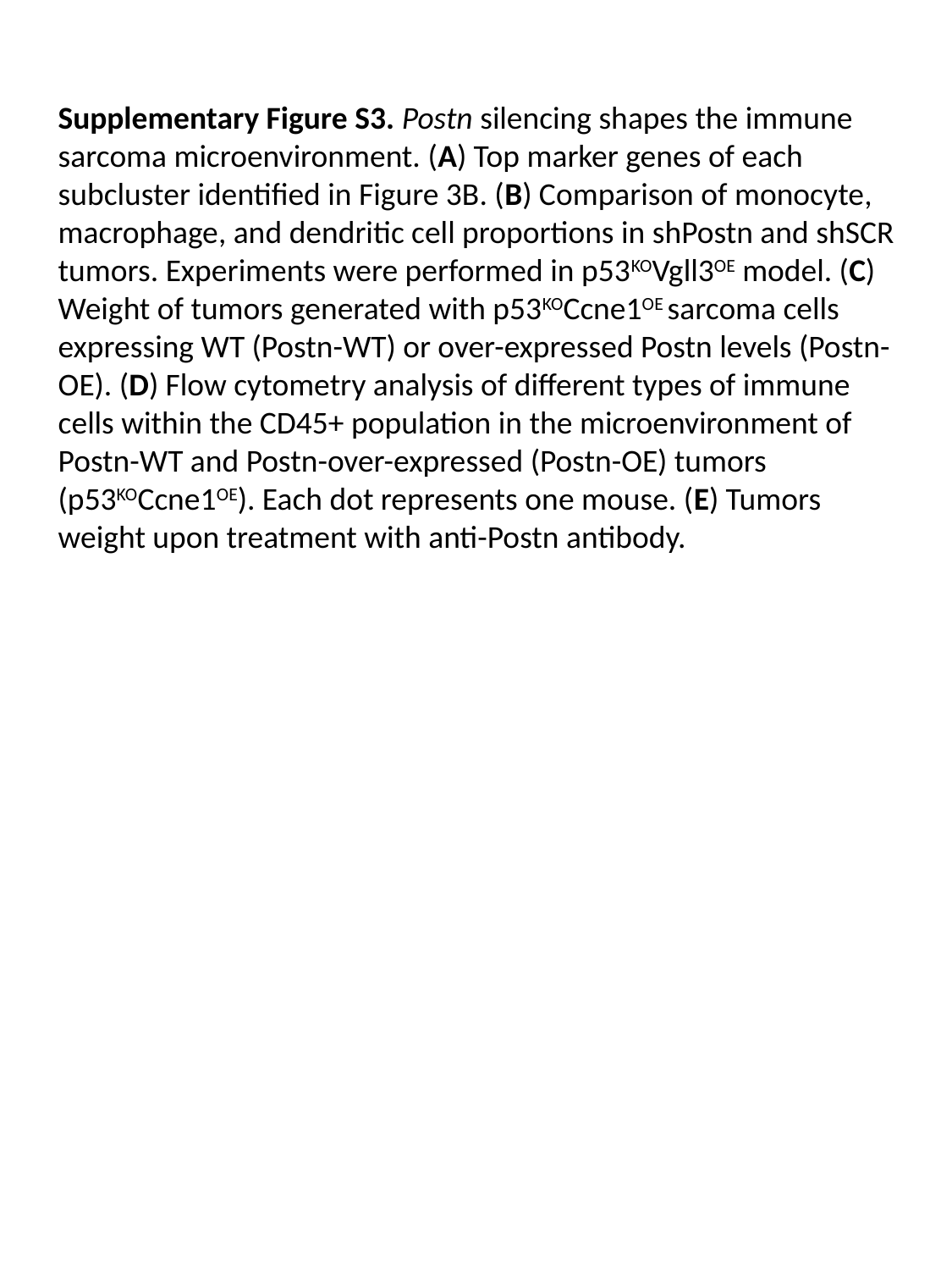

Supplementary Figure S3. Postn silencing shapes the immune sarcoma microenvironment. (A) Top marker genes of each subcluster identified in Figure 3B. (B) Comparison of monocyte, macrophage, and dendritic cell proportions in shPostn and shSCR tumors. Experiments were performed in p53KOVgll3OE model. (C) Weight of tumors generated with p53KOCcne1OE sarcoma cells expressing WT (Postn-WT) or over-expressed Postn levels (Postn-OE). (D) Flow cytometry analysis of different types of immune cells within the CD45+ population in the microenvironment of Postn-WT and Postn-over-expressed (Postn-OE) tumors (p53KOCcne1OE). Each dot represents one mouse. (E) Tumors weight upon treatment with anti-Postn antibody.
