# Supplementary material for: Periostin Promotes Sarcoma Growth by Promoting Tumor-Associated Macrophage Migration and Differentiation
Source: Cancer Res Commun. 2025 Dec 26;5(12):2224–35. doi: 10.1158/2767-9764.CRC-25-0301 (PMC12740715; doi:10.1158/2767-9764.CRC-25-0301)
Supplement: Supplementary Figure S4 — Figure S4. Differential expression of macrophage-related genes in BMDM vs BMDM cultured with recombinant POSTN (rPOSTN). [file crc-25-0301_supplementary_figure_s4_suppsf4.pptx]

## Slide 1
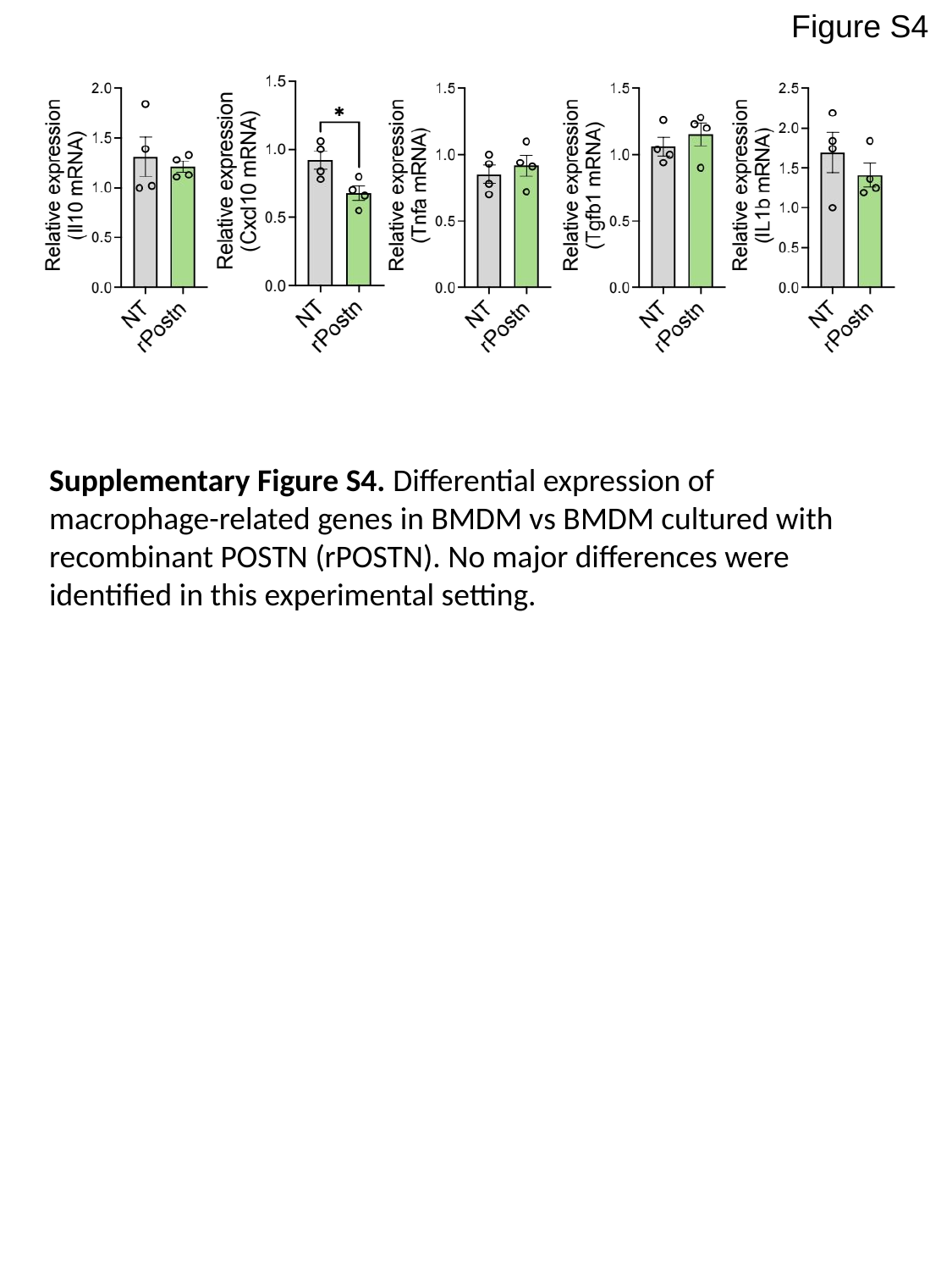

Figure S4
Supplementary Figure S4. Differential expression of macrophage-related genes in BMDM vs BMDM cultured with recombinant POSTN (rPOSTN). No major differences were identified in this experimental setting.
